# 11β-HSD1 plays a critical role in trabecular bone loss associated with systemic glucocorticoid therapy

**DOI:** 10.1186/s13075-019-1972-1

**Published:** 2019-08-16

**Authors:** C. G. Fenton, C. L. Doig, S. Fareed, A. Naylor, A. P. Morrell, O. Addison, C. Wehmeyer, C. D. Buckley, M. S. Cooper, G. G. Lavery, K. Raza, R. S. Hardy

**Affiliations:** 1 Institute of Inflammation and Ageing, University of Birmingham, Birmingham, UK; 2 Institute of Metabolism and Systems Research, University of Birmingham, Birmingham, UK; 3 Aston Institute of Materials Research, Aston University, Birmingham, UK; 4 Institute of Clinical Sciences, University of Birmingham, Birmingham, UK; 5 Faculty of Medicine and Dentistry, University of Alberta, Edmonton, Canada; 6 ANZAC Research Institute, University of Sydney, Sydney, Australia; 7 Sandwell and West Birmingham Hospitals NHS Trust, Birmingham, UK

**Keywords:** Glucocorticoids, Osteoporosis, 11β-HSD1, Trabecular bone

## Abstract

**Background:**

Despite their efficacy in the treatment of chronic inflammation, the prolonged application of therapeutic glucocorticoids (GCs) is limited by significant systemic side effects including glucocorticoid-induced osteoporosis (GIOP). 11β-Hydroxysteroid dehydrogenase type 1 (11β-HSD1) is a bi-directional enzyme that primarily activates GCs in vivo, regulating tissue-specific exposure to active GC. We aimed to determine the contribution of 11β-HSD1 to GIOP.

**Methods:**

Wild type (WT) and 11β-HSD1 knockout (KO) mice were treated with corticosterone (100 μg/ml, 0.66% ethanol) or vehicle (0.66% ethanol) in drinking water over 4 weeks (six animals per group). Bone parameters were assessed by micro-CT, sub-micron absorption tomography and serum markers of bone metabolism. Osteoblast and osteoclast gene expression was assessed by quantitative RT-PCR.

**Results:**

Wild type mice receiving corticosterone developed marked trabecular bone loss with reduced bone volume to tissue volume (BV/TV), trabecular thickness (Tb.Th) and trabecular number (Tb.N). Histomorphometric analysis revealed a dramatic reduction in osteoblast numbers. This was matched by a significant reduction in the serum marker of osteoblast bone formation P1NP and gene expression of the osteoblast markers *Alp* and *Bglap*. In contrast, 11β-HSD1 KO mice receiving corticosterone demonstrated almost complete protection from trabecular bone loss, with partial protection from the decrease in osteoblast numbers and markers of bone formation relative to WT counterparts receiving corticosterone.

**Conclusions:**

This study demonstrates that 11β-HSD1 plays a critical role in GIOP, mediating GC suppression of anabolic bone formation and reduced bone volume secondary to a decrease in osteoblast numbers. This raises the intriguing possibility that therapeutic inhibitors of 11β-HSD1 may be effective in preventing GIOP in patients receiving therapeutic steroids.

**Electronic supplementary material:**

The online version of this article (10.1186/s13075-019-1972-1) contains supplementary material, which is available to authorized users.

## Introduction

Therapeutic glucocorticoids (GCs) show marked efficacy in the treatment of chronic inflammatory conditions. Unfortunately, prolonged exposure to GCs results in severe adverse metabolic side effects including osteoporosis, insulin resistance and obesity, severely limiting their long-term therapeutic application [1–3]. Glucocorticoid-induced osteoporosis (GIOP) is common in patients receiving therapeutic GCs with 30–50% of patients developing decreased bone mineral density and increased fracture risk within 6 months [4–6]. Several mechanisms have been proposed whereby GCs cause loss of bone mineral density and deterioration in bone architecture. Chief amongst these is the direct inhibition of the osteoid-forming osteoblasts within bone, as evidenced by a marked and rapid suppression of serum P1NP and osteocalcin in patients receiving the therapeutic GC prednisolone [7]. In addition, GCs cause increased bone resorption by supporting the survival, differentiation and activation of osteoclasts in vivo [8–12]. Additional mechanisms whereby GCs drive bone loss include the suppression of anabolic sex steroids as well as calcium and vitamin D metabolism and induction of myopathy that collectively contribute to systemic bone loss [13, 14].

11β-Hydroxysteroid dehydrogenase type 1 (11β-HSD1) is a bi-directional enzyme that, in the presence of the NADPH-generating enzyme H6PDH, primarily activates GCs (cortisone to cortisol in humans, 11-dehydrocorticosterone to corticosterone in mice) in vivo and determines their tissue-specific exposure [15]. In response to therapeutic glucocorticoids, such as hydrocortisol and prednisolone, renal inactivation competes with hepatic reactivation of steroids, providing both active and inactive glucocorticoid substrates in the circulation for tissue-specific metabolism by 11β-HSD1 [16, 17]. Pre-receptor metabolism of GCs by this enzyme has been shown to be critical in mediating insulin resistance, obesity, skin thinning and hepatic steatosis in mice following exposure to both active and inactive GCs [18]. This is in part mediated through renal inactivation of active GCs by 11β-hydroxysteroid dehydrogenase type 2 (11β-HSD2), which are then recycled within peripheral target tissues expressing 11β-HSD1.

Currently, the contribution of 11β-HSD1 to GIOP is poorly understood despite its expression being reported in primary osteoblasts and bone, where it is potently upregulated by inflammation [19–23]. In this study, we employed a murine model of exogenous oral corticosterone delivery, known to closely mimic the kinetics of clinical GC therapy, in wild type (WT) and global 11β-HSD1 knockout (KO) mice to delineate the contribution of 11β-HSD1 to GIOP, and demonstrate its critical role in mediating the effects of therapeutic GCs on bone [24].

## Materials and methods

### 11β-HSD1 KO mouse model

Experiments were carried out at the University of Birmingham, UK (project licence number P51102987), following strict guidelines governed by the UK Animal (Scientific Procedures) Act 1986 and were approved by the local ethics committee (BERSC: Birmingham Ethical Review Subcommittee). 11β-HSD1 KO mice were generated as previously described through crossing HSD11B1 floxed mice with the ZP3-Cre expressing strain to achieve germline deletion of 11β-HSD1 [25]. Nine-week-old male WT or 11β-HSD1 KO littermate mice on a C57BL/6 J background had ad libitum access to standard chow and drinking water supplemented with either corticosterone (Cort) (100 μg/mL, 0.66% ethanol), or vehicle (0.66% ethanol) for 4 weeks (six animals per group, 24 animals in total). Treatments were replaced twice weekly. At the end of the experiment, 13-week-old animals were culled by cervical dislocation following a cardiac bleed under terminal anaesthetic and tissues excised, weighed and fixed in 4% formalin or snap-frozen in liquid nitrogen for later analyses.

### Analysis of mRNA abundance

Expression of specific mRNAs was determined using TaqMan® Gene Expression Assays (Thermo Fisher Scientific, Loughborough, UK). RNA was extracted from homogenised tibia. Briefly, whole tibias were removed from the hind limb ensuring complete removal of soft tissue under a dissection microscope. The heads of bone were removed at the metaphysis, and the bone marrow was flushed with a syringe. The diaphysis of the tibia was powdered in liquid nitrogen in a sterilised pestle and mortar. mRNA isolation was then performed on the resulting homogenate using an innuPREP RNA Mini Kit (Analytikjena, Cambridge, UK) as per the manufacturer’s instructions. Aliquots containing 1 μg of RNA were then reverse transcribed using random hexamers according to the manufacturer’s protocol (4311235, Multiscribe™, Thermo Fisher Scientific) to generate cDNA. The levels of murine 11β-HSD1 (*Hsd11b1*), RUNX2 (*Runx2*), OPG (*Tnfrsf11b*), RANKL (*Tnfsf11*), osteocalcin (*Bglap*), cathepsin K (*Ctsk*), alkaline phosphatase (*Alp*) and sclerostin (*Sost*) were assessed to determine expression of genes that define osteoblasts and osteoclasts and contribute to the balance of bone metabolism. Gene expression was determined using species-specific probe sets for real-time PCR on an ABI7500 system (Applied Biosystems, Warrington, UK). Final reactions contained 2X TaqMan PCR mastermix (Life Technologies), 200 nmol TaqMan probe and 25–50 ng cDNA. The abundance of specific mRNAs in a sample was normalised to that of 18S RNA. Data were obtained as Ct values and used to determine ΔCt values (Ct target − Ct 18S). Data were expressed as arbitrary units using the following transformation: [arbitrary units (AU) = 1000 × (2^−Δct^)].

### 11β-HSD1 activity of tibia tissue

Ex vivo tibia biopsies were placed in a culture medium containing 100 nmol/l of 11-dehydrocorticosterone (11-DHC) (to measure oxo-reductase/activation activity) along with tritiated [^3^H] tracer amounts of 11-DHC. Steroids were extracted using dichloromethane and separated by thin-layer chromatography using ethanol:chloroform (8:92) as the mobile phase. Thin-layer chromatography plates were analysed by a Bioscan imager (Bioscan, Washington, DC, USA) and the fractional conversion of steroids was calculated. The protein concentration was assessed by a 96-well assay kit (Bio-Rad). Results were expressed as picomole product/per milligramme of protein/hour, and experiments were performed in triplicate.

### Analysis of corticosterone, P1NP and CTX by ELISA

Serum was collected from mice by cardiac puncture under terminal anaesthetic. Briefly, whole blood was left at room temperature for 30 min prior to centrifugation for 20 min at 12,000 rpm. Serum was aspirated and stored at − 80 °C prior to analysis. Unbound, serum-free corticosterone levels were measured using a commercially available sandwich ELISA designed to specifically detect active (but not inactive 11DHC) steroid (cat no: KGE009, R&D systems, Abingdon, UK). Serum was analysed in accordance with the manufacturer’s instructions and data expressed as nanogrammes per millilitre (ng/ml). Serum P1NP was determined using a commercially available sandwich ELISA (cat no: AC-33F1, Immunodiagnostic Systems, Tyne & Wear, UK) in accordance with the manufacturer’s instructions and data expressed as ng/ml. Serum CTX-1 was determined using a commercially available sandwich ELISA (cat no: AC-06F1, Immunodiagnostic Systems, Tyne & Wear, UK) in accordance with the manufacturer’s instructions and data expressed as units per microlitre.

### Static histomorphometry

Static histomorphometry was performed by the skelet.AL Skeletal Analysis Laboratories. Briefly, lumbar vertebrae 3 and 4 were fixed in 10% neutral buffered formalin, decalcified in EDTA and embedded in paraffin, and 3-μm sections were cut using a Leica Microsystems microtome (Leica Microsystems, Milton Keynes, UK). The sections were stained with either haematoxylin and eosin or tartrate-resistant acid phosphatise (TRAP) to identify osteoclasts and counterstained with Gill’s haematoxylin. The sections were examined by light microscopy (Leica Microsystems). The number of osteoblasts and osteoclasts per millimetre were measured on 6.5 mm of the corticoendosteal surfaces, starting 0.25 mm from the growth plate using the Osteomeasure analysis software (Osteometrics, Decatur, GA, USA).

### Micro-CT morphometry analysis

Formalin-fixed tibiae from 13-week-old mice were scanned using a Skyscan 1172 X-ray microtomograph at 60 kV/167 μA with a 0.5-mm aluminium filter. Images were obtained at a 5-μm resolution with a rotation step of 0.45°. NRecon software was used to reconstruct the images. Trabecular and cortical bone parameters were analysed using CTAn Skyscan software: regions of interest (ROI) were selected by drawing around trabecular or cortical bone regions for each cross-sectional slice; the tibia and bone architecture was determined by quantifying trabecular and cortical bone parameters using CTAn software. Trabecular bones 1.35 mm in length (200 sections) were selected for trabecular bone analysis at the metaphyseal region near the growth plate. Extent was determined by the length of trabecular bone growth in each sample, which was calculated by multiplying slice number by pixel size of scanned image (13.5 μm). Meshlab software was used to process 3D meshes of tibiae and calculate trabecular bone volume to tissue volume (BV/TV), trabecular number (Tb.N), trabecular separation (Tb.Sp) and trabecular thickness (Tb.Th).

### Synchrotron sub-micron absorption tomography

Mice tibiae were examined on the Diamond Manchester Imaging Branch I13-2 beamline at the UK’s national synchrotron facility, Diamond Light Source (Harwell, UK). Whole bones were centrally mounted on a rotation-translation stage. A defocused polychromatic incident X-ray source (pink beam) was used to irradiate the entire sample. A PCO.edge 5.5 camera system containing a sCMOS sensor was positioned behind the sample to collect an X-ray absorption image. A × 4 objective lens was positioned in front of the camera sensor to provide a resolution of 0.81 μm and a total field of view of 2.1 mm horizontally and 1.8 mm vertically. Each measurement consisted of 2500 projections, recorded over an angular range of 360° with an irradiation time of 100 ms per projection. Full 3D reconstruction was performed using in house I-13 software following identification of the centre of rotation in a single orthogonal image from the mid-diaphysis to the region immediately below the proximal epiphysis line. The reconstructed volumes were analysed in software package Aviso®, where osteocyte lacunae were rendered and thresholded consistently for analysis of pore volume and morphology.

### Statistical analysis

Statistical significance was defined as *p* < 0.05 (*p < 0.05; **p < 0.01; ***p < 0.001) using either an unpaired Student *t* test or two-way ANOVA with a Bonferroni correction where a Gaussian distribution is identified (determined by both Kolmogorov-Smirnov and Shapiro-Wilk test), or a non-parametric Kruskal-Wallis test with Dunn’s Multiple Comparison where it is absent.

## Results

### Oral corticosterone induces GC excess in wild type and 11β-HSD1 KO animals

**Fig. 1:**
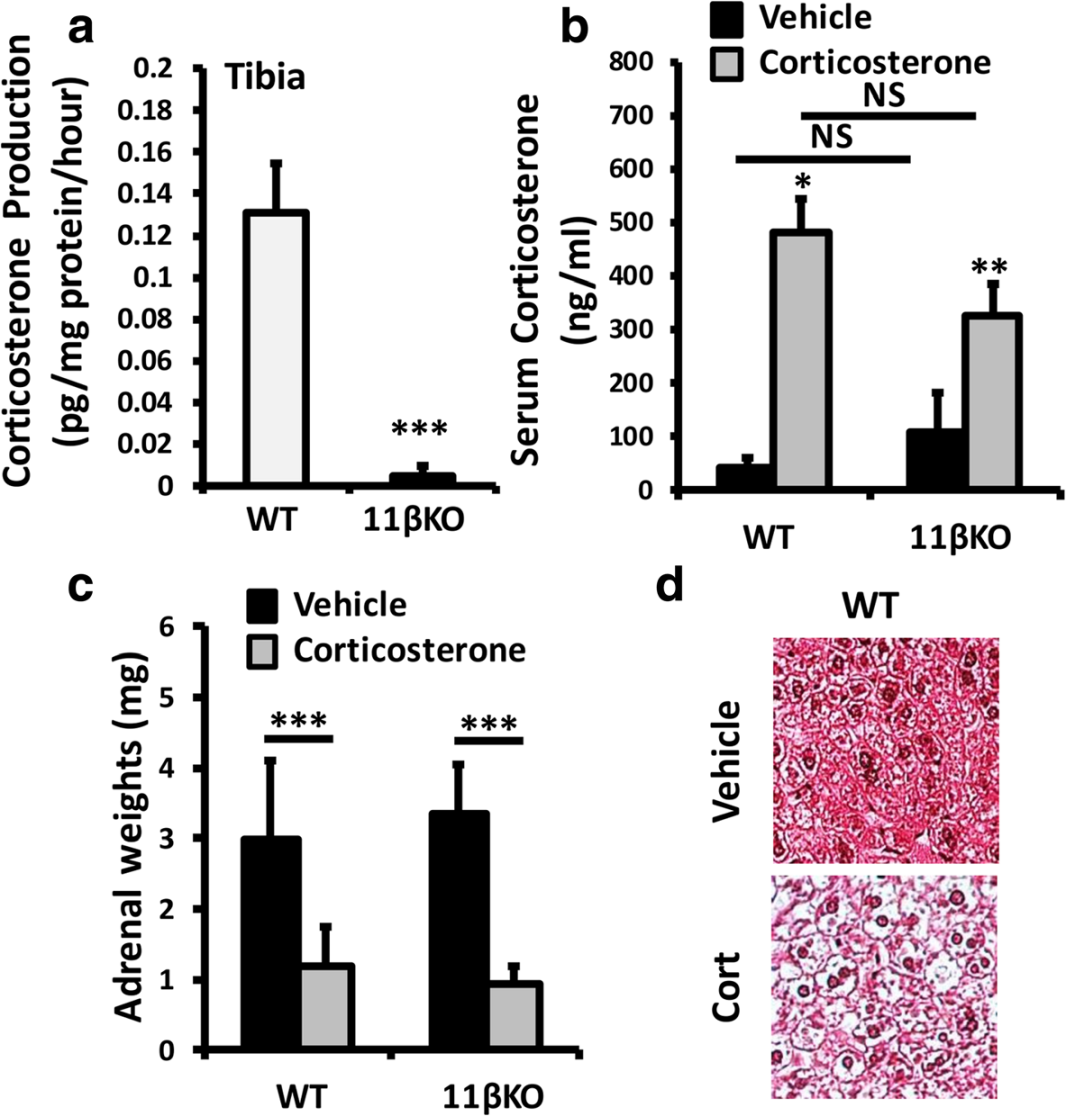
**a** Corticosterone generation in tibia ex vivo biopsies isolated from WT and 11β-HSD1 KO mice determined by scanning thin-layer chromatography. **b** Serum corticosterone levels determined by ELISA in WT and 11β-HSD1 KO receiving either vehicle or oral corticosterone (100 μg/ml). **c** Adrenal weights (mg) from WT and 11β-HSD1 KO mice receiving either vehicle or oral corticosterone (100 μg/ml) and **d** representative paraffin-embedded sections of the liver taken from WT mice receiving either vehicle or oral corticosterone (100 μg/ml) (× 20), stained with haematoxylin and eosin. Values are expressed as mean ± standard error of six animals per group. Statistical significance was determined using two-way ANOVA with a Bonferroni correction. *p < 0.05, **p < 0.01, ***p < 0.001

Nine-week-old C57BL/6 WT and global 11β-HSD1 KO mice received drinking water containing either vehicle or corticosterone at 100 μg/ml for 4 weeks. Deletion of 11β-HSD1 and inhibition of corticosterone generation in the bones of 11β-HSD1 KO mice was confirmed in ex vivo tibia biopsies, where corticosterone generation from DHC was significantly abrogated in 11β-HSD1 KO mice compared to WT animals (Fig. 1a). Expression of *H6pd* (the gene encoding the NADPH cofactor-generating enzyme H6PDH) required for 11β-HSD1 steroid activation was highly expressed and did not change in tibiae, across groups (Additional file 1: Figure S1a).

Evidence of circulating GC excess was determined by measuring midnight (within normal active phase) serum corticosterone levels. Serum levels of corticosterone were significantly increased in both WT and 11β-HSD1 KO animals receiving corticosterone in drinking water relative to those receiving vehicle (WT, 41.2 ± 12.3 ng/ml versus WT + Cort, 479.6 ± 76.1 ng/ml, *p* < 0.01; 11β-HSD1 KO, 108.2 ± 72.2 ng/ml versus 11β-HSD1 KO + Cort, 329.5 ± 51.6 ng/ml, *p* < 0.05) (Fig. 1b) (Additional file 2). Serum levels were not significantly different between WT and 11β-HSD1 KO animals receiving corticosterone. Increased systemic exposure to corticosterone was evidenced by the marked suppression of adrenal weights in both WT and 11β-HSD1 KO animals receiving corticosterone and the onset of hepatic steatosis in WT animals (Fig. 1c, d). These data confirm that oral administration of corticosterone in drinking water at 100 μg/ml is sufficient to induce circulating GC excess in both WT and 11β-HSD1 KO animals.

### 11β-HSD1 KO showed protection from corticosterone-induced trabecular bone

**Fig. 2:**
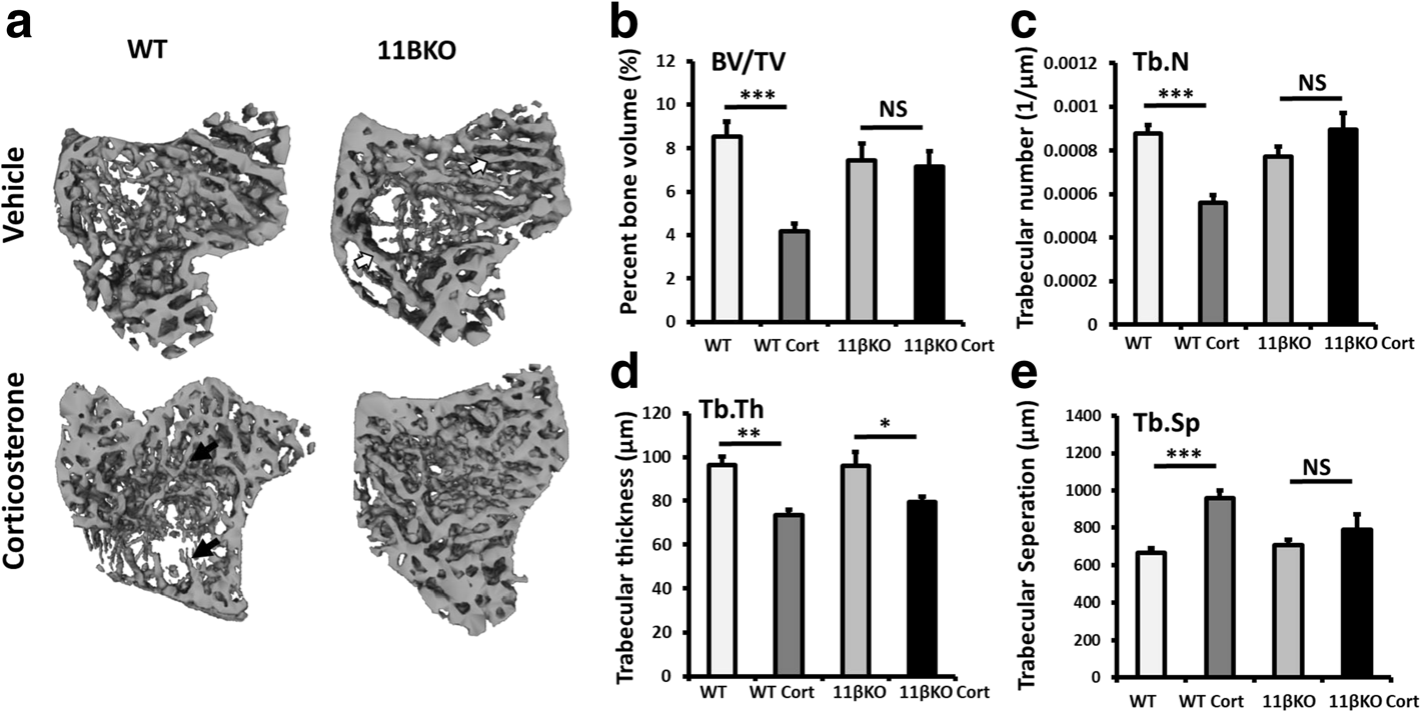
**a** Representative images of 3D reconstructions of tibia trabecular bone using micro-CT from WT and 11β-HSD1 KO receiving either vehicle or oral corticosterone (100 μg/ml). **b** Bone volume to tissue volume (BV/TV), **c** trabecular number (Tb.N), **d** trabecular thickness (Tb.Th) and **e** trabecular separation (Tb.Sp) determined by Meshlab software analysis of micro-CT in WT and 11β-HSD1 KO receiving either vehicle or oral corticosterone (100 μg/ml). Values are expressed as mean ± standard error of six animals per group. Statistical significance was determined using two-way ANOVA with a Bonferroni correction. *p < 0.05, **p < 0.01, ***p < 0.001. Black arrows represent regions of mesh surface trabecular thinning

To determine the role of 11β-HSD1 in GIOP, we generated 3D trabecular meshes from the tibia following micro-CT using Meshlab software (Fig. 2a). Analysis of 3D trabecular meshes demonstrated that trabecular bone volume to tissue volume (BV/TV), trabecular number (Tb.N), trabecular separation (Tb.Sp) and trabecular thickness (Tb.Th) were identical between vehicle-treated WT and 11β-HSD1 KO animals (Fig. 2b–e). Following oral corticosterone administration over 4 weeks, a significant reduction in trabecular bone parameters was identified in WT animals (BV/TV: WT, 8.5% ± 0.66 vs WT + Cort, 4.2% ± 0.38, *p* < 0.001; Tb.N: WT, 0.0009 1/μm ± 0.00004 vs WT + Cort, 0.0006 1/μm ± 0.00004, *p* < 0.01; Tb.Th: WT, 96.5 μm ± 3.8 vs WT + Cort, 73.5 μm ± 3.5, *p* < 0.01; Tb.Sp: WT, 664 μm ± 27 vs WT + Cort, 959 μm ± 31, *p* < 0.01) (Fig. 2b–e). In contrast, 11β-HSD1 KO mice receiving corticosterone were protected from this reduction in trabecular BV/TV, Tb.N and Tb.Sp relative to vehicle-treated controls (BV/TV: 11β-HSD1 KO, 7.5% ± 0.76 vs 11β-HSD1 KO + Cort, 7.2% ± 0.71, NS; Tb.N: 11β-HSD1 KO, 0.0008 1/μm ± 0.00004 vs 11β-HSD1 KO + Cort, 0.0009 1/μm ± 0.00008, NS; Tb.Sp: 11β-HSD1 KO, 706.9 μm ± 28, NS vs 11β-HSD1 KO + Cort, 789 μm ± 61, NS) (Fig. 2b, c). In contrast, 11β-HSD1 KO animals were not protected from suppressed Tb.Th in response to corticosterone with a significant reduction identified in these animals relative to vehicle-treated controls (Tb.Th: 11β-HSD1 KO 95.8 μm ± 5.2 vs11β-HSD1 KO + Cort, 79.4 μm ± 3.1, *p* < 0.05) (Fig. 2d). Micro-CT analysis of cortical bone from 3D cortical bone reconstructions revealed no significant differences in cortical thickness (Crt.T), cortical cross-sectional area (Crt.A), endosteal medullary area (Med.A), periosteal perimeter (Per.P) or cortical lacunae properties between WT and 11β-HSD1 KO animals (Additional file 1: Figure S1a-g).

These data indicate that treatment with oral corticosterone at 100 μg/ml in drinking water for 4 weeks is sufficient to induce marked trabecular bone loss at the tibia of WT C57BL/6 animals. In contrast, animals with deletion of 11β-HSD1 demonstrate significant protection against the bone-wasting effects of oral corticosterone in trabecular bone.

### GC-induced suppression of osteoblast numbers and bone formation markers was blunted in 11β-HSD1-KO mice

**Fig. 3:**
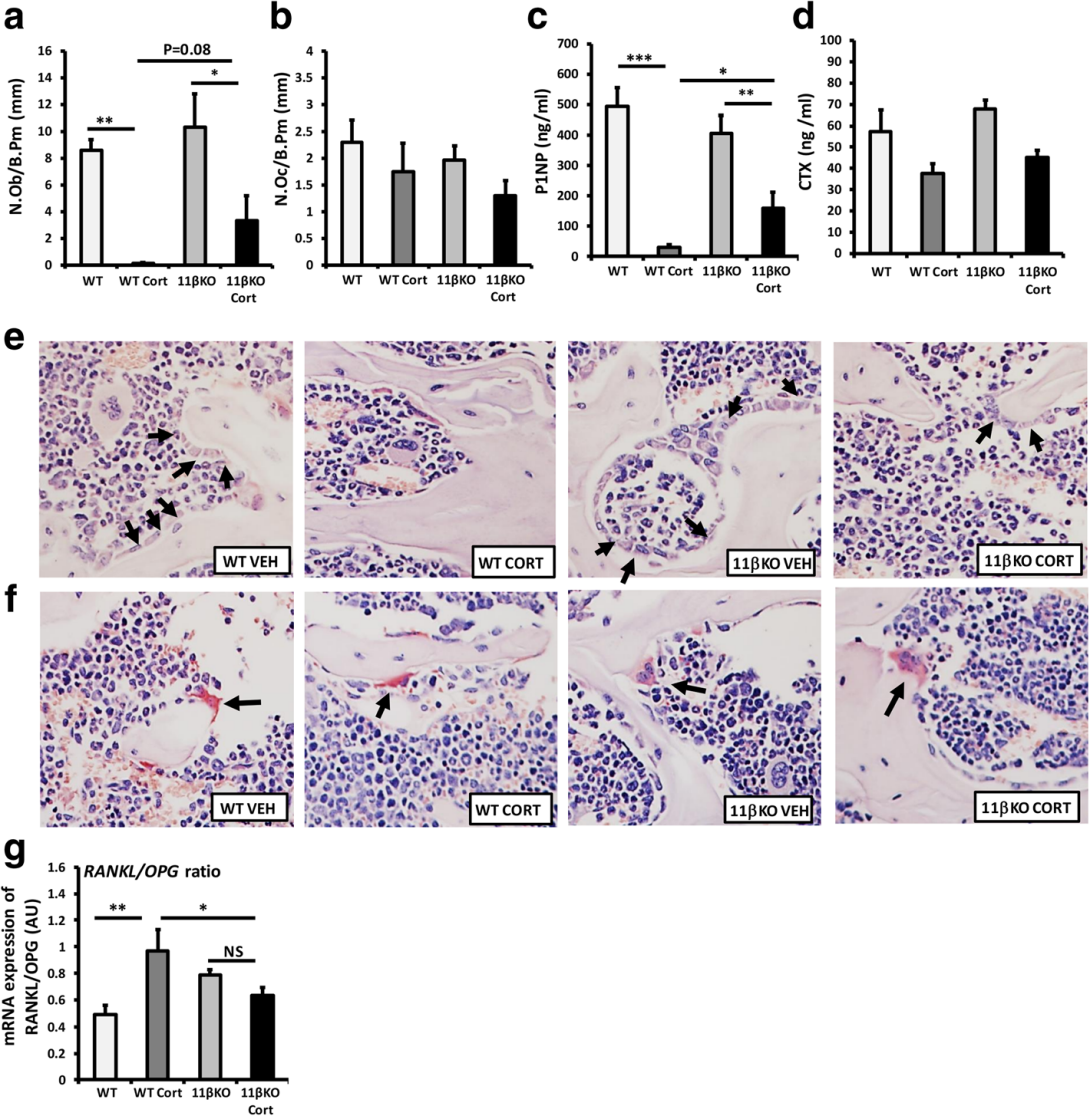
Histomorphometric analysis of numbers of (**a**) osteoblasts (N.Ob/B.Pm) and (**b**) osteoclasts (N.Oc/B.Pm) at the bone perimeter per square millimetre from vertebrae L3 and L4. **c** Serum P1NP (ng/ml) (**d**) and serum CTX-1 (ng/ml) were determined by ELISA in WT and 11β-HSD1 KO mice receiving either vehicle or oral corticosterone (100 μg/ml). **e** Representative images of osteoblasts and **f** representative images of osteoclasts on trabecular bone surface. **g** The ratio of RANKL/OPG gene expression in the tibia from WT and 11β-HSD1 KO mice receiving either vehicle or oral corticosterone (100 μg/ml) was determined by quantitative RT-PCR. Values are expressed as mean ± standard error of six animals per group. Statistical significance was determined using two-way ANOVA with a Bonferroni correction. *p < 0.05, **p < 0.01, ***p < 0.001. Black arrows indicate osteoblasts and osteoclasts

Bone metabolism is tightly regulated by the balance between osteoblast-mediated bone formations and osteoclast bone resorption. Analysis of bone osteoblast and osteoclast numbers and serum biomarkers of bone formation (procollagen type 1 amino-terminal propeptide (P1NP)) and bone resorption (degradation products from C-terminal telopeptides of type I collagen (CTX-1)) was performed by histomorphometry and ELISA respectively to ascertain the impact of oral corticosterone on these cell populations. A dramatic decrease in osteoblast numbers per bone perimeter (Ob.N./B.pm) was readily apparent in WT mice receiving oral corticosterone relative to controls, with an almost total absence of osteoblasts (WT, 8.5 + 1.7 mm, versus WT + Cort, 0.1 + 0.07 mm; *p* < 0.001)(Fig. 3a, e). This was partially abrogated in 11β-HSD1 KO mice receiving corticosterone, where osteoblast numbers were detectable, despite a significant suppression (11β-HSD1 KO, 10.3 + 2.9, versus 11β-HSD1 KO + Cort, 3.3 + 2.1 ng/ml; *p* < 0.05). These results were closely mirrored by a comparable dramatic decrease in serum P1NP in WT mice receiving oral corticosterone (WT, 494.2 + 67, versus WT + Cort, 31.3 + 2.1 ng/ml; *p* < 0.00) that was also partially abrogated in 11β-HSD1 KO mice (11β-HSD1 KO, 405.7 + 69.4, versus 11β-HSD1 KO + Cort, 158.6 + 55.1 ng/ml; *p* < 0.01) (Fig. 3c). Serum levels of P1NP were significantly higher in 11β-HSD1 KO mice receiving corticosterone than in WT counterparts (WT + Cort, 31.3 + 2.1, versus 11β-HSD1 KO + Cort, 158.6 + 55.1 ng/ml; *p* < 0.05).

In contrast to osteoblasts, no significant changes in osteoclast numbers per bone perimeter (Oc.N./B.pm) or in serum measures of osteoclast activity determined by CTX-1 were observed in WT and 11β-HSD1 KO mice receiving GCs (Fig. 3b, d, f). The ratio of RANKL/OPG gene expression was examined as a critical regulator of osteoclast formation and activation in ex vivo tibia biopsies (Fig. 3). A significant increase in the RANKL/OPG ratio was apparent in WT mice receiving oral corticosterone (1.9-fold; *p* < 0.01). 11β-HSD1 KO mice were protected from this increased ratio in response to oral corticosterone with no significant change in expression relative to 11β-HSD1 KO mice receiving vehicle and a significantly lower ratio compared to WT animals receiving GCs (Fig. 3e).

**Fig. 4:**
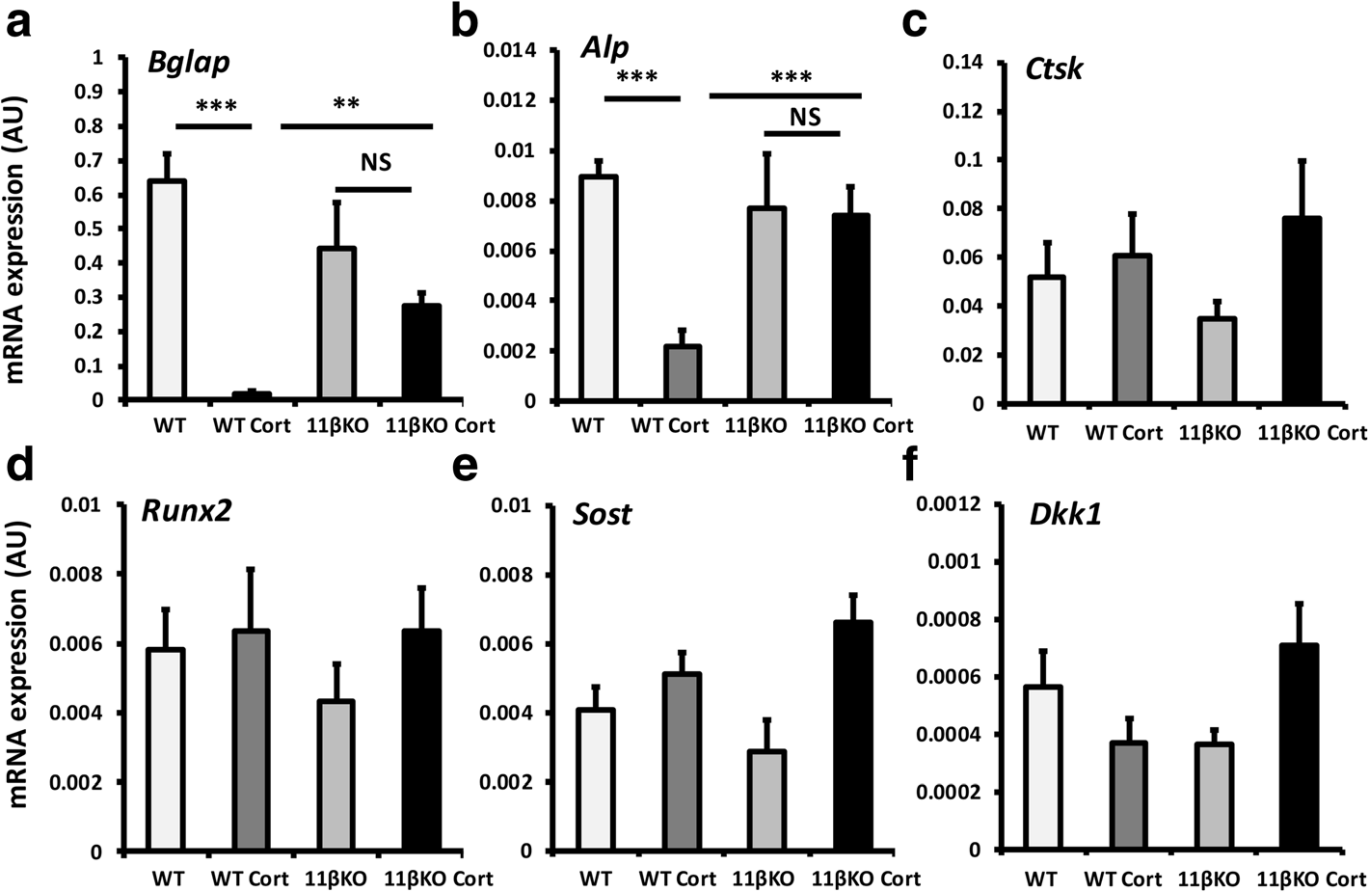
**a–f** Gene expression (AU) of *Bglap*, *Alp*, *Ctsk*, *Runx2*, *Sost* and *Dkk1* in tibias taken from WT and 11β-HSD1 KO receiving either vehicle or oral corticosterone (100 μg/ml) determined by quantitative RT-PCR. Values are expressed as mean ± standard error of six animals per group. Statistical significance was determined using two-way ANOVA with a Bonferroni correction. *p < 0.05, **p < 0.01, ***p < 0.001

Analysis of markers of mature osteoblast gene expression in whole ex vivo biopsies of tibia was determined by quantitative RT-PCR. In WT mice, the osteoblast markers *Bglap* and *Alp* were significantly reduced following administration of oral corticosterone (*Bglap*, 33-fold; *p* < 0.0001, *Alp*, 4-fold; *p* < 0.01) (Fig. 4a, b). In contrast, 11β-HSD1 KO mice showed significant protection from the suppression of *Bglap* with no significant change in expression, whilst the suppression of *Alp* was completely abrogated following administration of oral corticosterone (Fig. 4b). mRNA expression of the osteoclast marker *Ctsk*, the master regulator of osteoblast differentiation *Runx2* and the negative regulators of osteoblast differentiation, *Sost* and *Dkk1*, were not altered in either WT or 11β-HSD1 KO mice receiving oral corticosteroids (Fig. 4c–f).

Taken together, these data strongly indicate that the bone loss identified in WT mice receiving corticosterone is characterised by a profound suppression in osteoblast numbers and bone formation, and a shift in the resorption/formation ratio that would favour net bone loss. This appears to be partially dependant on 11β-HSD1 activity, where 11β-HSD1 KO animals show significant, but not complete protection from the suppression in osteoblast activity.

## Discussion

Despite important systemic side effect, GCs continue to be routinely employed in the management of chronic inflammatory diseases such as rheumatoid arthritis. In this study, we show for the first time that pre-receptor metabolism of exogenously administered GCs by the enzyme 11β-HSD1 is a key component mediating bone loss in a murine model of GIOP. Here, following administration of active glucocorticoids such as corticosterone, renal and hepatic metabolism ensures an equilibrium between active and inactive glucocorticoid substrates, which are then available for tissue-specific pre-receptor activation by the 11β-HSD enzymes [17, 18]. Previously, the GC receptor (GR) has been shown to be critical in mediating GIOP in mouse models of GC excess with the targeted deletion of GR in both osteoblasts and osteoclasts being shown to be protective [26, 27].

We used a model of oral administration of corticosterone in drinking water to delineate the precise contribution of pre-receptor GC metabolism by 11β-HSD1 to GIOP using a global KO model. Previously, this model of exogenous GC excess has been shown to result in a consistent diurnal exposure pattern, closely mimicking the kinetics of clinical GC therapy [24]. Of note, systemic and renal inactivation of glucocorticoid by 11β-HSD2 has been shown to be unaffected in the global 11β-HSD1 KO mouse in response to corticosterone [28].

Both WT and 11β-HSD1 KO mice treated with exogenous corticosterone showed signs of corticosterone excess with significantly elevated levels of the serum-free steroid and marked suppression of adrenal weights relative to untreated controls. Furthermore, WT mice developed hepatic steatosis in response to corticosterone treatments in line with classical presentations of GC excess previously reposted in human and mouse models [18, 29].

Analysis of trabecular bone in the tibias of WT animals revealed a significant reduction in all trabecular bone parameters following addition of corticosterone. These data are supportive of a systemic GC-induced bone loss in WT C57BL/6 mice in response to corticosterone in drinking water at 100 μg/ml for 4 weeks.

Similar studies have reported a robust decrease in bone mass in response to therapeutic GCs such as prednisolone in C57BL/6 mice [30, 31]. These studies identify a significant decrease in trabecular and cortical content at the tibia in response to subcutaneous prednisolone pellets over 28 days. The bone loss phenotype observed in our model is less marked, but is broadly consistent with this, with evidence of early trabecular bone loss at the tibia.

In vivo, GCs have been shown to potently suppress osteoblast-mediated bone formation by increasing both apoptosis and autophagy [32–35]. Certainly in this model, we observed a dramatic suppression of osteoblast numbers in trabecular bone of wild type mice treated with corticosterone, with a robust suppression of P1NP as a marker of systemic bone formation and a marked suppression of mature osteoblast markers including osteocalcin and alkaline phosphatase. Together, these data suggest that this model of GC excess is comparable to those previously reported and suitable to examine the role of 11β-HSD1.

Importantly, mice with a global deletion of 11β-HSD1 demonstrated a significant protection from trabecular bone loss at the tibia following administration of exogenous corticosterone in drinking water. This approached full protection from reductions in BV/TV, trabecular number and trabecular separation and conferred a partial protection from reduced trabecular thickness. This protective effect appeared to be mediated through a resistance to GC-induced suppression of bone formation in osteoblasts, with a partial preservation of trabecular osteoblast numbers, increased serum P1NP levels and elevated expression of the mature osteoblast markers, osteocalcin and alkaline phosphatase in 11β-HSD1 KO animals relative to WT counterparts receiving corticosterone. Further experiments in these animals might utilise delivery of inactive steroid metabolites such as DHC to assess 11β-HSD1-mediated activation and tissue-specific targeting without interference from residual active corticosterone to examine its role in vivo.

Previous studies examining the overexpression of 11β-HSD2 targeted to osteoblasts and osteocytes in mice, mediating complete GC signalling blockade in these cells, have identified a phenotype characterised by reduced cranial ossification and bone mineral density [36, 37]. These studies demonstrate that GC signalling is required for normal osteoblast and osteocyte maturation and function. Deletion of 11β-HSD1 did not reproduce these findings in our study, suggesting that basal GC signalling mediated by free circulating active GCs is sufficient to mediate normal bone development.

In contrast, targeted blockade of GC signalling in osteoblasts and osteocytes using either the overexpression of 11β-HSD2 or the inhibition of GR dimerization is able to prevent GIOP in murine models of GC excess [26, 32]. We see similar findings in the 11β-HSD1 KO mouse suggesting that, whilst total levels of the active steroid are increased in our model, they are insufficient to induce trabecular bone loss in the absence of 11β-HSD1 GC activation.

These prior studies provide compelling evidence that the deleterious actions of GCs are mediated directly through osteoblasts via an increase in osteoblast apoptosis and autophagy. Whilst our studies do not address in which cell type deletion of 11β-HSD1 is mediating protection from GIOP, previous studies demonstrating robust expression of 11β-HSD1 in vivo and vitro strongly indicate that 11β-HSD1 expression within osteoblasts is likely to mediate the protection reported in our global 11β-HSD1 KO mice [20, 22, 26, 32]. However, the possibility that 11β-HSD1 within alternative cell populations such as osteoclasts cannot be discounted. Regardless, better characterisation of the 11β-HSD1-expressing cell subtypes that mediate protection may prove beneficial in the future where targeting of therapeutic inhibitors of 11β-HSD1 may be of interest to more effectively prevent GIOP.

In this model, we chose oral administration of corticosterone at 100 μg/ml to initiate GC excess in male C57BL/6 mice and so cannot extrapolate these findings to female animals. This dose of corticosterone was selected due to the strong evidence of diurnal exposure patterns, which closely mimic that seen in patients following oral therapeutic GC administration [24]. Other methods such as subcutaneous pellets result in a continuous steady delivery of GC. Whilst this allows for the better control of drug release, it may be less representative of delivery regimes in patients.

## Conclusions

`For the first time, this study demonstrates that 11β-HSD1 plays a critical role in mediating the detrimental actions of exogenous therapeutic corticosterone administration on bone and that its targeted deletion is able to ameliorate GIOP in this murine model. This raises the intriguing possibility that therapeutic inhibitors of 11β-HSD1 may be effective in preventing GIOP in patients receiving therapeutic steroids.

## Additional files

Additional file 1: **Figure S1.** (a), Representative images of 3D reconstructions of tibia cortical bone using micro-CT from WT and 11β-HSD1 KO receiving either vehicle or oral corticosterone (100 μg/ml). (b), Cortical cross-sectional thickness (Crt.Cs.T), (c), cortical cross-sectional area (Crt.Cs.A), (d) endosteal medullary area (Med.A) and (e) periosteal perimeter (Per.P) determined by Meshlab software analysis of micro-CT in WT and 11β-HSD1 KO receiving either vehicle or oral corticosterone (100 μg/ml). (f), Quantification of osteoblast lacunae in murine cortical bone collected at I-13 using pink beam, count time 100 ms, rotations 2500. Full 3D reconstruction was performed using in house I-13 script following identification of centre of rotation in a single orthogonal slice. Volume rendering of osteocyte lacunae was performed in Aviso® prior to pore analysis of Volume3d and Area^3^d. (f), Average pore area (μm^3^), (g) lacunae number within a 100μm^3^ region of interest in WT and 11β-HSD1 KO receiving either vehicle or oral corticosterone (100 μg/ml). Values are expressed as mean ± standard error of three animals per group. Statistical significance was determined using one way ANOVA with a Tukey’s post hoc analysis. Values are expressed as mean ± standard error of six animals per group. Statistical significance was determined using two way ANOVA with a Bonferroni correction. (TIFF 1190 kb)

Additional file 2: **Figure S2.** (a), Gene expression (AU) of H6pd determined by quantitative RT-PCR in WT and 11β-HSD1 KO receiving either vehicle or oral corticosterone (100 mg/ml). Values are expressed as mean ± standard error of six animals per group. Statistical significance was determined using two way ANOVA with a Bonferroni correction. (TIFF 316 kb)

## Data Availability

All data generated or analysed during this study are included in this published article [and its supplementary information files].

## Abbreviations

11-DHC: 11-Dehydrocorticosterone
11β-HSD1: 11β-Hydroxysteroid dehydrogenase type 1
BV/TV: Trabecular bone volume to tissue volume
Cort: Corticosterone
Crt.CS.A: Cortical cross-sectional area
Crt.Cs.T: Cortical cross-sectional thickness
GCs: Glucocorticoids
GIOP: Glucocorticoid-induced osteoporosis
KO: Knockout
Med.A: Endosteal medullary area
P1NP: Procollagen type 1 amino-terminal propeptide
Per.P: Periosteal perimeter
Tb.N: Trabecular number
Tb.Th: Trabecular thickness
WT: Wild type
