# Supplementary figures and images for: 11β-HSD1 plays a critical role in trabecular bone loss associated with systemic glucocorticoid therapy
Source: Arthritis Res Ther. 2019 Aug 16;21:188. doi: 10.1186/s13075-019-1972-1 (PMC6698000; doi:10.1186/s13075-019-1972-1)

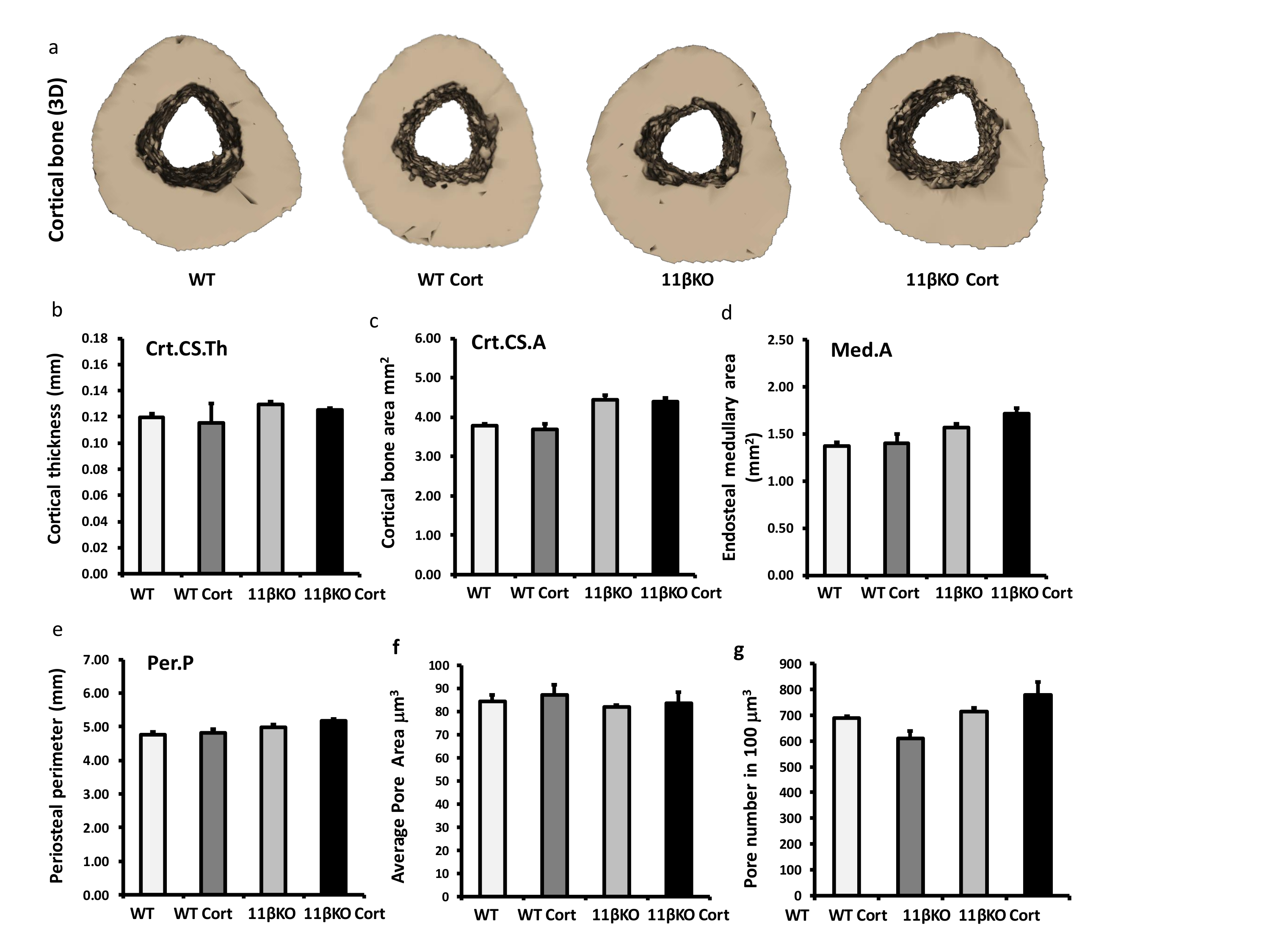

Supplement: Supplementary file 1 — Figure S1. (a), Representative images of 3D reconstructions of tibia cortical bone using micro-CT from WT and 11β-HSD1 KO receiving either vehicle or oral corticosterone (100 μg/ml). (b), Cortical cross-sectional thickness (Crt.Cs.T), (c), cortical cross-sectional area (Crt.Cs.A), (d) endosteal medullary area (Med.A) and (e) periosteal perimeter (Per.P) determined by Meshlab software analysis of micro-CT in WT and 11β-HSD1 KO receiving either vehicle or oral corticosterone (100 μg/ml). (f), Quantification of osteoblast lacunae in murine cortical bone collected at I-13 using pink beam, count time 100 ms, rotations 2500. Full 3D reconstruction was performed using in house I-13 script following identification of centre of rotation in a single orthogonal slice. Volume rendering of osteocyte lacunae was performed in Aviso® prior to pore analysis of Volume3d and Area3d. (f), Average pore area (μm3), (g) lacunae number within a 100μm3 region of interest in WT and 11β-HSD1 KO receiving either vehicle or oral corticosterone (100 μg/ml). Values are expressed as mean ± standard error of three animals per group. Statistical significance was determined using one way ANOVA with a Tukey’s post hoc analysis. Values are expressed as mean ± standard error of six animals per group. Statistical significance was determined using two way ANOVA with a Bonferroni correction. (TIFF 1190 kb) [file 13075_2019_1972_MOESM1_ESM.tiff]

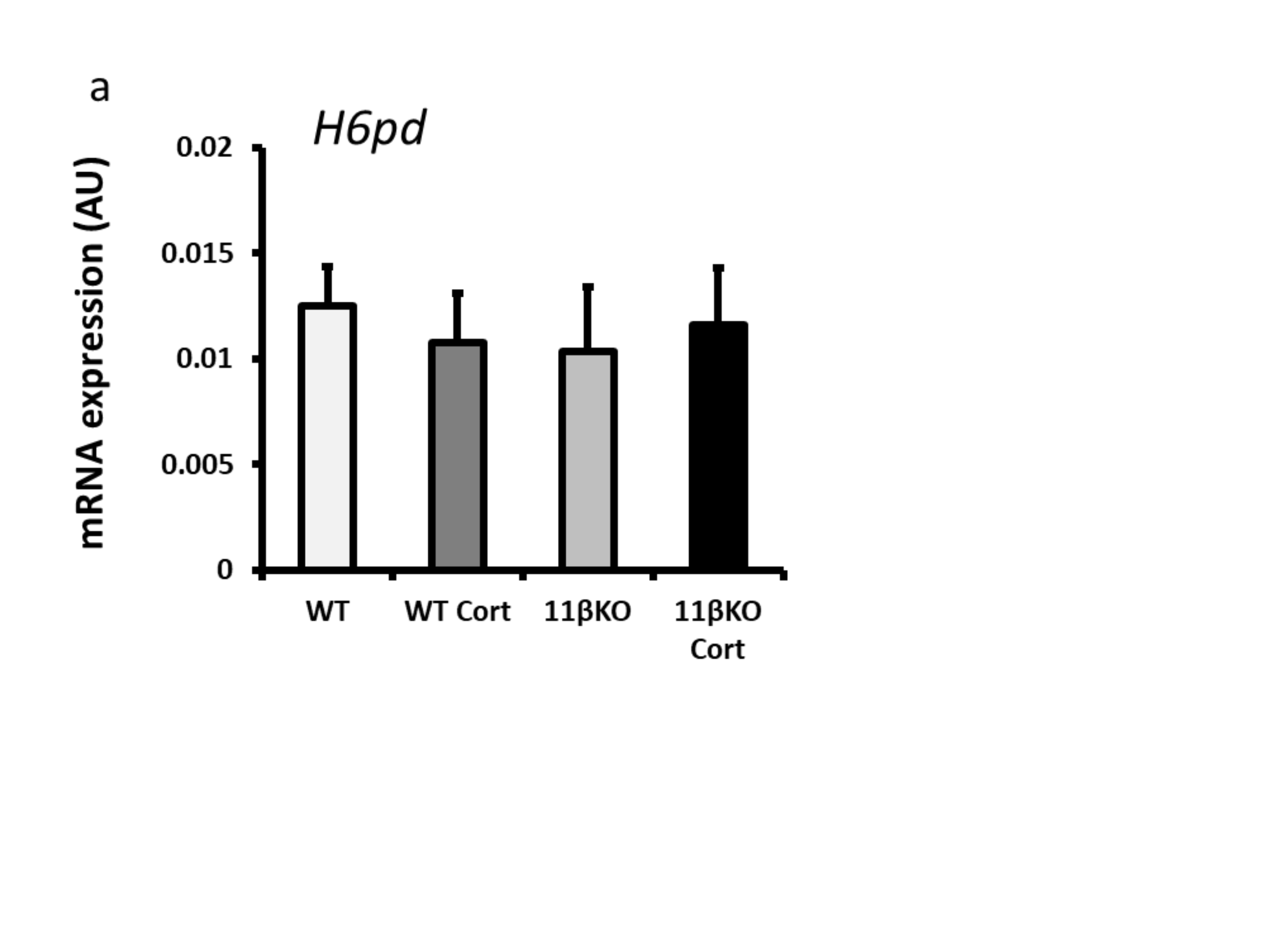

Supplement: Supplementary file 2 — Figure S2. (a), Gene expression (AU) of H6pd determined by quantitative RT-PCR in WT and 11β-HSD1 KO receiving either vehicle or oral corticosterone (100 mg/ml). Values are expressed as mean ± standard error of six animals per group. Statistical significance was determined using two way ANOVA with a Bonferroni correction. (TIFF 316 kb) [file 13075_2019_1972_MOESM2_ESM.tiff]
